# The effects of allospecific mitochondrial genome on the fitness of northern redbelly dace (*Chrosomus eos*)

**DOI:** 10.1002/ece3.3922

**Published:** 2018-02-19

**Authors:** Bernard Angers, Christelle Leung, Romain Vétil, Léo Deremiens, Roland Vergilino

**Affiliations:** ^1^ Department of Biological Sciences Université de Montréal Montreal QC Canada

**Keywords:** allospecific mitochondrial genome, *Chrosomus eos*, cybrid, mitochondrial introgression, northern redbelly dace

## Abstract

Instantaneous mitochondrial introgression events allow the disentangling of the effects of hybridization from those of allospecific mtDNA. Such process frequently occurred in the fish *Chrosomus eos*, resulting in cybrid individuals composed of a *C. eos* nuclear genome but with a *C. neogaeus* mtDNA. This provides a valuable model to address the fundamental question: How well do introgressed individuals perform in their native environment? We infer where de novo production of cybrids occurred to discriminate native environments from those colonized by cybrids in 25 sites from two regions (West‐Qc and East‐Qc) in Quebec (Canada). We then compared the relative abundance of wild types and cybrids as a measure integrating both fitness and de novo production of cybrids. According to mtDNA variation, 12 introgression events are required to explain the diversity of cybrids. Five cybrid lineages could not be associated with in situ introgression events. This includes one haplotype carried by 93% of the cybrids expected to have colonized West‐Qc. These cybrids also displayed a nearly complete allopatric distribution with wild types. We still inferred de novo production of cybrids at seven sites, that accounted for 70% of the cybrids in East‐Qc. Wild‐type and cybrid individuals coexist in all East‐Qc sites while cybrids were less abundant. Allopatry of cybrids restricted to the postglacial expansion suggests the existence of higher fitness for cybrids in specific conditions, allowing for the colonization of different environments and expanding the species’ range. However, allospecific mtDNA does not provide a higher fitness to cybrids in their native environment compared to wild types, making the success of an introgressed lineage uncertain.

## 1 INTRODUCTION

Hybridization and its impacts on fitness are of major importance in evolution and conservation biology (Allendorf, Leary, Spruell, & Wenburg, 2001; Dowling & Secor, 1997; Lewontin & Birch, 1966; Mavárez & Linares, 2008; Rhymer & Simberloff, 1996; Seehausen, 2006; Stebbins, 1959, 1985). One particular process that can result from hybridization is mitochondrial introgression. Mitochondria are involved in various cellular processes essential to the survival of individuals, such as energetic metabolism, apoptosis, and signaling pathways (Le Bras, Clément, Pervaiz, & Brenner, 2005). Protein complexes of the respiratory chain that allow the production of energy by mitochondria are encoded by both the mitochondrial (mtDNA) and the nuclear genomes (nucDNA). A strong co‐evolution between these genomes is expected to maintain the highly specific interactions between protein subunits required to be fully operational (Burton, Pereira, & Barreto, 2013; Osada & Akashi, 2012). Processes like mitochondrial introgression that break up the allele combinations of co‐adapted genes from mtDNA and nucDNA are therefore expected to result in outbreeding depression (Edmands & Burton, 1999; Ellison & Burton, 2008; McKenzie, Chiotis, Pinkert, & Trounce, 2003).

However, mitochondrial introgression has been reported in various organisms across taxonomic groups (Alves, Melo‐Ferreira, Freitas, & Boursot, 2008; Boratyński et al., 2011; Goddard & Schultz, 1993; Irwin, Rubtsov, & Panov, 2009; Liu et al., 2010; Llopart, Herrig, Brud, & Stecklein, 2014; Marková, Dufresne, Manca, & Kotlík, 2013; McGuire et al., 2007; Ruedi, Smith, & Patton, 1997; Toews, Mandic, Richards, & Irwin, 2014; Wang & Wang, 2014; Wilson & Bernatchez, 1998; Zieliński et al., 2013). Introgression may allow rapid physiological and life history trait adjustment and adaption (*i.e*., adaptive introgression) to cope with specific environments or climatic changes (Boratyński et al., 2011; Llopart et al., 2014; Toews et al., 2014). Although mitochondrial introgression has been observed in many animals, it remains anecdotal for most species reported. This has limited the study of the genetic, ecological, and environmental factors underlying these events. The relative fitness between wild types and introgressed types has only been determined for a few examples (e.g., Montooth, Meiklejohn, Abt, & Rand, 2010; Muhlfeld et al., 2009; Rand, Fry, & Sheldahl, 2006). In addition, the frequency of mtDNA haplotypes transferred to a receiving species is particularly important to understanding the potential adaptive processes underlying introgressive hybridization (Rheindt & Edwards, 2011; Toews & Brelsford, 2012).

Mitochondrial introgression generally results from asymmetric hybridizations and repeated backcrossing and is therefore a slow process lasting several generations (Rieseberg & Wendel 1993). This phenomenon may also occur in a single generation in some hybrid complexes (Goddard & Schultz, 1993; Yamada et al., 2015). Hybrids of these complexes are perpetuating lineages with their own evolutionary fate. They can occasionally produce individuals in which a diploid nuclear genome and mtDNA from different species are brought together in a single generation. Such instantaneous mitochondrial introgression events allow the disentangling of the effects of hybridization from those of allospecific mtDNA.

**Figure 1:**
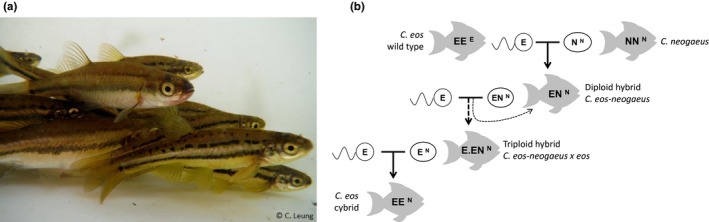
Instantaneous mitochondrial introgression in the fish *Chrosomus eos*. (a) Individuals of the complex *Chrosomus eos‐neogaeus* including *C. eos* and hybrids. (b) Hybridization between a male *C. eos* and a female *C. neogaeus* results in diploid hybrids. These all‐female hybrids reproduce clonally by gynogenesis; sperm is only required to trigger egg development. Occasional incorporation of the genome of *C. eos* sperm results in triploid hybrids. Triploid hybrids can produce eggs with a haploid *C. eos* genome but a *C. neogaeus* mtDNA. Instantaneous mitochondrial introgression occurs when such an egg is fertilized by the *C. eos* sperm, leading to de novo production of cybrids. E and N refer to nuclear genome of *C. eos* and *C. neogaeus*, respectively, superscript to mitochondrial genome

In the northern redbelly dace (*Chrosomus eos*), individuals can harbor either *C. eos* or *C. neogaeus* mtDNA, referred to hereafter as wild types and cybrids, respectively (Figure 1a). Instantaneous mitochondrial introgression resulting in de novo production of cybrids (Figure 1b) is possible due to the presence of all‐female hybrids *C. eos–neogaeus* (Goddard, Dawley, & Dowling, 1989). These hybrids reproduce clonally by gynogenesis; sperm of either *C. eos* or *C. neogaeus* is required, but only to trigger the development of the unreduced eggs (Goddard, Megwinoff, Wessner, & Giaimo, 1998). However, a high proportion of triploid hybrids may occur when the nuclear genome of *C. eos* sperm is incorporated in unreduced hybrid eggs (Leung & Angers, 2018). Triploid hybrids are expected to occasionally produce eggs with a haploid *C. eos* genome and *C. neogaeus* mtDNA (Goddard & Schultz, 1993). Fertilization of such an egg by a *C. eos* haploid sperm reconstitutes the diploid nuclear genome of *C. eos* but with a *C. neogaeus* mitochondrial genome and results in an instantaneous mitochondrial introgression (Angers & Schlosser, 2007; Binet & Angers, 2005; Goddard & Schultz, 1993).

Cybrids produced de novo inherit of a combination of nuclear and mitochondrial genomes that have not co‐evolved since speciation time (ca. 5 Myears, Deremiens, Schwartz, Angers, Glémet, & Angers, 2015). Moreover, they must compete with *C. eos* wild type that are expected to be well adapted to environmental conditions. These *Chrosomus eos* cybrids can therefore provide a valuable model for addressing a fundamental question about mitochondrial introgression: How well do introgressed individuals perform through time in their native environment compared to wild types?

This study aimed to assess the effects of allospecific mtDNA *C. neogaeus* on the fitness of *C. eos* in their native environment. More specifically, we compared the long‐term demography of *C. eos* cybrids to that of the wild types from which they occurred. Once produced, cybrids can reproduce sexually as wild types do. However, de novo production of cybrids represents an additional input of individuals that can demographically favor cybrids. On the one hand, cybrids are expected to exclude wild types if they display fitness similar or higher to that of wild types, as theoretically demonstrated by Barron, Lawson, and Jensen (2016). On the other hand, a lower fitness of cybrids can be demographically compensated by de novo production of individuals so that both cybrids and wild types can coexist in sympatry. To test these predictions, we determined the relative abundance of wild types and cybrids in a survey of 25 *C. eos* populations from southern Quebec (Canada). The relative abundance integrates the long‐term demography by taking into account both fitness and the additional input of cybrids.

Determining where de novo production of cybrids occurred is of primary importance when assessing the fitness of cybrids in their native environment. This is particularly relevant in geographic contexts strongly modeled by postglacial expansion such as northeastern North America (April, Hanner, Dion‐Côté, & Bernatchez, 2013; Bernatchez & Wilson, 1998; Gagnon & Angers, 2006), because cybrids of a given site may also originate from postglacial colonization. We thus inferred where de novo production of cybrids occurred to discriminate between the native environments and those colonized by cybrids during the postglacial expansion. At a given site, we inferred de novo production of cybrids when cybrids and one sympatric hybrid lineage (the *C. neogaeus* mtDNA donor) shared the same mtDNA sequence. In the absence of sympatric hybrids or if the mitochondrial haplotype of cybrids did not match with that of sympatric hybrids, we assumed that an introgression event occurred prior to postglacial colonization or that hybrid lineage that gave rise to the cybrid was extinct at this site.

We performed the survey in two regions (West‐Qc and East‐Qc) known to display contrasting patterns of *C. eos‐neogaeus* hybrid diversity. In West‐Qc, one hybrid lineage is widespread throughout this region resulting from postglacial colonization (Angers & Schlosser, 2007). In East‐Qc, multiple hybridization events occurred in situ and lineages displayed a narrow geographic distribution (Vergilino, Leung, & Angers, 2016) and may therefore represent different sources of cybrids.

## 2 MATERIAL AND METHODS

### 2.1 Prevalence of wild types and cybrids

**Figure 2:**
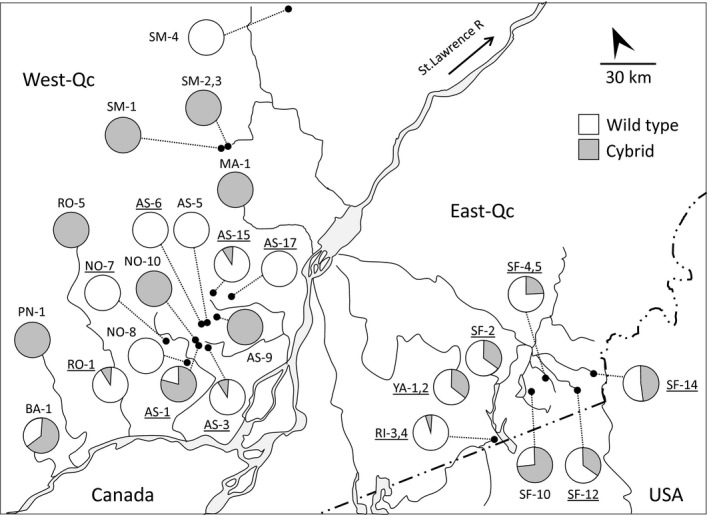
Map of the southern Quebec (Canada) and geographic location of the sites analyzed for this study. The relative abundance of *Chrosomus eos* wild type and cybrid was indicated for sites from West‐Qc (*n* = 18) and East‐Qc (*n* = 7). The underlined names refer to the presence of hybrids at those sites

A total of 664 individuals visually identified as *Chrosomus eos* (New, 1962) were collected from 18 sites in West‐Qc and seven sites in East‐Qc (Table 1, Figure 2) in southern Quebec (Canada). The sampling of wild type and cybrid individuals was random considering as they could not be visually discriminated. The sampling was performed under institutional animal care guidelines (permit #13‐084 delivered by the *Université de Montréal*) and conforms to the mandatory guidelines of the Canadian Council on Animal Care. Sampling permits were provided by the Quebec Ministry of Natural Resources and Wildlife (MRNF).

**Table 1:** Characteristics of the sampled sites. Geographic coordinates, relative abundance of wild‐type and cybrid individuals per site, sample size (*n*), and Nei's gene diversity

Site	Latitude (N)	Longitude (W)	*Wild type*	*Cybrid*	*n*	Diversity
*West‐Qc*						
BA‐1	45° 47′ 08″	75° 14′ 57″	0.32	0.68	25	0.44
PN‐1	46° 12′ 43″	75° 13′ 60″	0	1	8	0
RO‐1	45° 46′ 38″	74° 34′ 25″	0.85	0.15	13	0.27
RO‐5	46° 35′ 43′	74°33′ 48″	0	1	8	0
NO‐7	45° 56′ 37″	74° 11′ 37″	1	0	10	0
NO‐8	45° 52′ 31″	74° 08′ 44″	1	0	12	0
NO‐10	45° 55′ 32″	74° 03′ 51″	0	1	18	0
AS‐1	45° 55′ 01″	74° 04′ 22″	0.19	0.81	113	0.32
AS‐3	45° 54′ 53″	74° 01′ 41″	0.89	0.11	28	0.2
AS‐5	45° 59′ 17″	74° 00′ 24″	1	0	25	0
AS‐6	45° 59′ 22″	74° 00′ 18″	1	0	10	0
AS‐9	45° 57′ 14″	73° 56′ 06″	0	1	12	0
AS‐15	46° 05′ 34″	73° 52′ 20″	0.92	0.08	12	0.17
AS‐17	46° 05′ 32″	73° 48′ 33″	1	0	47	0
MA‐1	46° 30′ 30″	73° 23′ 00″	0	1	12	0
SM‐1	46° 47′ 43″	73° 20′ 56″	0	1	12	0
SM‐2,3	46° 47′ 15″	73° 16′ 43″	0	1	12	0
SM‐4	47° 19′ 55″	72° 35′ 24″	1	0	8	0
Total			0.51	0.49	385	0.08 (0.50)
*East‐Qc*						
YA‐1,2	45° 23′ 23″	72° 27′ 01″	0.65	0.35	31	0.47
RI‐3,4	45° 04′ 09″	72° 21′ 12″	0.94	0.06	17	0.12
SF‐2	45° 21′ 17″	72° 13′ 05″	0.67	0.33	12	0.48
SF‐4,5	45° 14′ 01″	71° 54′ 28″	0.73	0.27	15	0.42
SF‐10	45° 12′ 37″	71° 56′ 10″	0.29	0.71	69	0.42
SF‐12	45° 07′ 48″	71° 40′ 22″	0.67	0.33	115	0.45
SF‐14	45° 11′ 04″	71° 33′ 13″	0.55	0.45	20	0.52
Total			0.64	0.36	279	0.41 (0.46)

We first genetically confirmed the identity of individuals using markers specific to the *C. eos* nucDNA according to Binet and Angers (2005). We then used a PCR‐RFLP‐based method to identify individuals as wild type or cybrid according to mtDNA. In a single PCR, we used two pairs of primers (CR‐eos and CR‐neogaeus; Table 2) to specifically amplify a *C. eos* (627 bp) or a *C. neogaeus* (620 bp) D‐loop segment. Amplicons were then digested using the restriction enzyme *Hinf I*, producing four fragments (11, 82, 105, and 429 bp) from the amplified segment of *C. eos* mtDNA (wild type), while the segment from *C. neogaeus* (cybrid) remained uncut. This PCR‐RFLP procedure allowed for the rapid survey of the large number of individuals included in this study.

**Table 2:** Primer pairs for mitochondrial segments. Primers sequence, annealing temperature (*T*_M_), and the length of the amplified segments in base pairs (bp)

MtDNA segment	Primers sequence (5′ ‐ 3′)	*T*_M_	Length (bp)
*RFLP identification*			
CR‐eos	CCTAGATATGGCTCCCAACAG	50	627
	ATAATGCGGATGGCTAACCC		
CR‐neogaeus	CTCAACTTTTTCCTTGACATAC	50	620
	ATAATGCGGATGGCTAACGG		
*SSCP survey*			
D‐loop	ACCCCTGGCTCCCAAAGC	52	315
	AACCGGTTGGTGGTCTCTTACT		
ND3	CCCAGGGAAAGATAATGAAC	54	200
	GAGAATTGCTACGAGGAA		
COI	CCAGTGTTAGCAGCCGGAAT	60	307
	GGGTGTCTACGTCTATGCC		
*Sequencing*			
ND3‐4L	CCCAGGGAAAGATAATGAAC	54	674
	TGGCTACTAGGAGTGCAAG		

We partitioned the diversity of mtDNA detected in *C. eos* (wild type or cybrid) by calculating the diversity overall sites (H_T_), the diversity within population (H_S_) using the average of Nei's gene diversity (H_E_), and diversity among populations (*F*_ST_). Estimations and tests between regions were performed using F‐stat v2.9.3 (Goudet, 2001).

### 2.2 Inference of in situ production of cybrids

We inferred the locations of de novo production of cybrids to discriminate between native environments and those founded during the postglacial expansion. A correspondence between the cybrid and sympatric hybrid lineage mtDNA was considered as a de novo production of cybrids and the site as a native environment of cybrids. The procedure was achieved in three steps: (1) a wide‐scale survey using the single strand conformation polymorphism method (SSCP; Orita, Suzuki, Sekiya, & Hayashi, 1989) to analyze a large number of individuals; (2) the identification of mtDNA haplotypes according to previous studies using a reference gene; and (3) the confirmation of the correspondence between cybrid and hybrid mtDNA detected at step 1 by sequencing a large and variable region of the mtDNA.

We first performed the survey of mitochondrial DNA diversity using three gene segments (D‐loop, ND3, and COI; Table 2) over a sample of 202 individuals previously identified as cybrids. In addition, 13 distinct hybrid lineages in sympatry with *C. eos* (either wild type or cybrid) characterized in previous studies (Leung, Breton, & Angers, 2016; Vergilino et al., 2016) were included in this survey as putative *C. neogaeus* mtDNA donors. Amplifications were carried out using the following conditions: one cycle of denaturation at 92°C for 30 s, 45 cycles of denaturation at 92°C for 30 s, annealing temperature for 20 s (Table 2), and extension at 68°C for 30 s, with a final extension at 68°C for 2 min. Denatured PCR products were discriminated on an 8% nondenaturing polyacrylamide gel (acrylamide ⁄ bisacrylamide 37.5:1) for 12 hr at 15 W at 4°C in TBE 1X following the SSCP method (SSCP; Orita et al., 1989).

We then assigned each of the different haplotypes recovered by SSCP to the haplotypes A, B, and F previously found by Angers and Schlosser (2007) and Mee and Taylor (2012). We sequenced a segment of the cytochrome oxidase I (COI) gene (Angers & Schlosser, 2007) and searched for sequence similarity in GenBank.

Finally, we confirmed the correspondence of *C. neogaeus* mitochondrial haplotypes between sympatric cybrids and hybrids by sequencing a mitochondrial segment (hereafter designed as ND3‐4L) encompassing a portion of the ND3 and ND4L genes and the tRNA‐Arg gene using the ND3‐4L primers (Table 2). We retrieved 522‐bp quality sequences that were aligned using the MUSCLE algorithm available with the MEGA7 software (Kumar, Stecher, & Tamura, 2016).

### 2.3 Relative abundance of wild types and cybrids

The relative abundance of wild type and cybrid individuals in a given environment was used as a measure integrating both fitness and demographic inputs of cybrids. More specifically, we assessed the co‐occurrence of wild types and cybrids according to the origin of cybrids (de novo or migrants) using the C‐score index (Stone & Roberts, 1990). The C‐score statistic calculates the number of sites for which wild types and cybrids never appear together. High C‐score values indicate an increasing degree of mutual exclusivity between biotypes. The observed degree of allopatry was tested against 999 random communities generated according to a null model as described by Jonsson (2001).

We also constructed Mantel correlograms for establishing the spatial distribution of wild types and cybrids. We used straight line distance and waterway distance between sites. We used F_ST_ values as dependent variables and a geographic distance matrix as an independent variable. The number of distance classes was determined according to Sturges’ rule, and Mantel statistics was tested with 999 permutations using the correction for multiple tests proposed by Holm (1979). C‐score and Mantel statistics were performed in R (R Development Core Team 2004) using the *vegan* package (Oksanen et al., 2015).

## 3 RESULTS

### 3.1 Distribution of wild types and cybrids

PCR‐RFLP identification of the 664 *C. eos* individuals performed at the mitochondrial level allowed the detection of 375 (56.5%) wild‐type and 289 (43.5%) cybrid individuals. Wild types were as abundant as cybrids in West‐Qc (50.9% vs. 49.1%; χ^2^ = 0.065; *p* = .799), but more abundant in East‐Qc (64.2% vs. 35.8%; χ^2^ = 7.918; *p* = .005; Table 1). Cybrids were detected in 12 of 18 sites in West‐Qc and in all the sites in East‐Qc (Figure 2). For a comparable regional diversity between West‐Qc (H_T_ = 0.50) and East‐Qc (H_T_ = 0.46), diversity within population is significantly lower (*p* = .017) in West‐Qc (H_S_ = 0.08) than in East‐Qc (H_S_ = 0.41). However, the diversity among populations is significantly higher (*p* = .009) in West‐Qc (*F*_ST_ = 0.72) than in East‐Qc (*F*_ST_ = 0.16). These results indicate a more heterogeneous distribution of wild types and cybrids in the West‐Qc region, where several *C. eos* populations are exclusively composed of wild type or cybrid individuals (Figure 2).

### 3.2 Large‐scale survey of cybrid diversity

**Figure 3:**
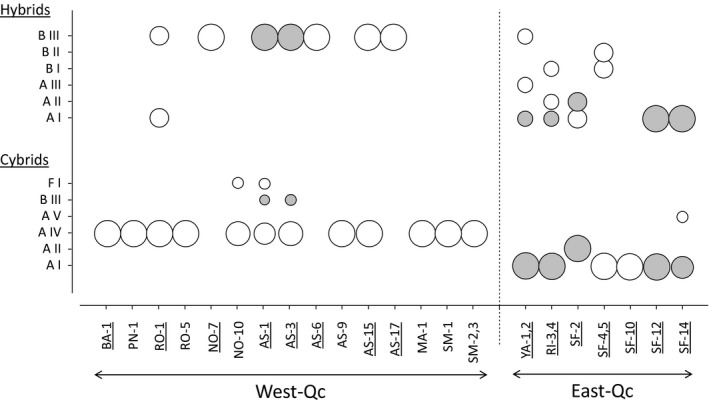
Relative abundance of the *C. neogaeus* haplotypes in cybrids. Circle's area corresponds to the proportion of haplotypes detected in cybrids. In hybrids, similar abundance was provided when several lineages were detected at a given site. Dark circles represent haplotypes shared between sympatric cybrids and hybrids. The underlined names refer to the presence of wild types at those sites

A total of six distinct haplotypes for *C. neogaeus* mitochondrial DNA were detected by combining variations in D‐loop, ND3, and COI segments detected by SSCP (Figure 3; Table 3) over 202 cybrid individuals from both regions. Searching for sequence similarity using a segment of the COI gene allows for recovering the three different groups of haplotypes A, B, and F (Angers & Schlosser, 2007; Mee & Taylor, 2012), including four closely related haplotypes in the group A. The haplotypes are designated using the letters of each cluster and a roman numeral (Figure 3; Table 3).

**Table 3:** Composition of the mitochondrial haplotypes. Identity of the SSCP conformers detected at the three segments (D‐loop, ND3, and COI) for *C. neogaeus* mtDNA in the different biotypes (cybrids and/or hybrids)

Haplotype	D‐loop	ND3	COI	Biotype
A I	A	A	A	Cybrids, hybrids
A II	B	A	A	Cybrids, hybrids
A III	A	A	B	Hybrids
A IV	A	B	A	Cybrids
A V	Null	A	A	Cybrids
B I	A	C	B	Hybrids
B II	A	C	A	Hybrids
B III	Null	C	B	Cybrids, hybrids
F I	C	D	C	Cybrids

The distribution of mtDNA in cybrids revealed a strong geographic break as none of their haplotypes is shared between regions. Most of the cybrids from West‐Qc were characterized by the haplotype A IV, while those from the East‐Qc region displayed the haplotype A I (Figure 3). This led to a high Nei's gene diversity for overall sites (H_E_ = 0.58). However, most populations displayed no haplotypic diversity. Only four populations revealed more than one haplotype, including site AS‐1 from West‐Qc that harbored three divergent haplotypes (A IV, B III, and F I).

The survey of the 13 hybrid lineages revealed a total of six haplotypes, with three closely related haplotypes for each of the groups A and B (Figure 3; Table 3). The distribution of hybrids diversity was similar to that observed in cybrids, as both regions displayed a different genetic composition: Haplotype B III was dominant in West‐Qc and haplotypes A, and to a lesser extent haplotype B, characterized hybrids from East‐Qc.

Cybrids displayed three haplotypes shared with sympatric hybrids (A I, A II, and B III; Figure 3). In West‐Qc, no correspondence between cybrid and hybrid haplotypes was detected, except for haplotype B III, which was present in very low abundance in cybrids at sites AS‐1 and AS‐3. In East‐Qc, in all sites but one (SF‐4,5), both cybrids and hybrids that are sympatric shared the same haplotype (A I at four sites and A II at one site). This suggests that de novo production of cybrids likely occurred multiple times from sympatric hybrids.

### 3.3 Inference of in situ production of cybrids

Correspondence between mtDNA haplotypes shared between sympatric cybrids and hybrids as putative donor was confirmed at the sequence level using the ND3‐4L segment (GenBank accession numbers [MG793359 to MG793378]). The sequences are designated by a lowercase letter when distinct sequences of a given SSCP haplotype were recovered. Cybrids without matching mtDNA in sympatric hybrid displayed distinct sequences at the ND3‐4L segment: haplotype A I‐b from SF‐4,5, A IV and F from AS‐1 and NO‐10, and A V from SF‐14 (Table 4).

**Table 4:** Sequence identity of *C. neogaeus* mtDNA based on ND3‐4L locus between cybrids and hybrids in sympatry. Haplotypes indicative of in situ formation of cybrids are shaded

Site	*C. eos* cybrids		Sympatric hybrids		
	SSCP Haplotype	ND3‐4L sequence	Lineages	SSCP Haplotype	ND3‐4L sequence
*West‐Qc*					
AS‐1	B III	B	B‐01	B III	B
AS‐1	A IV	A IV			
AS‐1	F I	F			
AS‐3	B III	B	B‐01	B III	B
NO‐10	A IV	A IV	—		
NO‐10	F I	F	—		
*East‐Qc*					
YA‐1,2	A I	A I‐a	A‐34	A I	A I‐a
YA‐1,2			A‐33	A III	
RI‐3,4	A I	A I‐a	A‐11	A I	A I‐a
RI‐3,4			A‐12	A II	
SF‐2	A II	A II^a^	A‐27	A II	A II^a^
SF‐2			A‐26	A I	
SF‐4,5	A I	A I‐b	B‐06	B II	
SF‐4,5			B‐07	B I	
SF‐10	A I	A I‐c	—		
SF‐12	A I	A I‐d	A‐18	A I	AI‐d
SF‐12			A‐19	A I	
SF‐14	A I	A I‐c	A‐06	A I	A I‐c
SF‐14	A V		A‐09	A I	

a Haplotype A II differed from haplotype A I at SSCP pattern, but displayed the same sequence at ND‐3,4 than A I‐c.

However, identical sequences were recovered from cybrids and one of their sympatric hybrid lineages at seven sites: two in West‐Qc and five in East‐Qc (Table 4). In West‐Qc, a correspondence for sequence B with hybrid lineage B‐01 was detected in two sites (sites AS‐1 and AS‐3). In East‐Qc, we detected correspondence for sequence A I‐a (hybrid lineage A‐34: site YA‐1,2; hybrid lineage A‐11: site RI‐3,4), sequence A I‐d (hybrid lineage A‐18: site SF‐12), sequence A I‐c (hybrid lineage A‐06: site SF‐14), and sequence A II (hybrid lineage A‐27: site SF‐2). Altogether, these results suggested in situ production of cybrids in seven sites.

### 3.4 Relative abundance of wild types and cybrids

In West‐Qc, 96% of the cybrids were not produced in situ and most of them (93%) belong to the A IV haplotype (Figure 3; Table 5). Wild types and cybrids are allopatric in six sites and seven sites, respectively, and were sympatric in only five of the 18 sites. Such a degree of observed allopatry is significantly different from that expected under a null model (C‐score = 42, *p* = .016). This distribution of wild types and cybrids in the West‐Qc region is not explained by either geographic or hydrologic proximity, as no significant spatial autocorrelations (*p* > .108) were observed for the straight line or waterway distances (data not shown). At the opposite, 69.9% of the cybrids from East‐Qc were produced de novo (Figure 3; Table 5). Distribution of wild types and cybrids in East‐Qc strongly contrasted with that of West‐Qc as both forms were found in sympatry in all sites (Figure 2). Moreover, wild types are more abundant than cybrids at all sites but one (SF‐10; Table 1).

**Table 5:** Mitochondrial diversity in the cybrid populations. Relative abundance of the different *C. neogaeus* haplotypes detected in 202 introgressed *C. eos* individuals. Asterisk refers to cybrids produced in situ

	A I	A II	A IV	A V	B III	F I	N
*West‐Qc*							
BA‐1	—	—	1	—	—	—	4
PN‐1	—	—	1	—	—	—	4
RO‐1	—	—	1	—	—	—	2
RO‐5	—	—	1	—	—	—	7
NO‐10	—	—	0.83	—	—	0.17	18
AS‐1	—	—	0.67	—	0.15*	0.18	91
AS‐3	—	—	0.67	—	0.33*	—	3
AS‐9	—	—	1	—	—	—	8
AS‐15	—	—	1	—	—	—	1
MA‐1	—	—	1	—	—	—	4
SM‐1	—	—	1	—	—	—	4
SM‐2,3	—	—	1	—	—	—	8
Total	—	—	0.93	—	0.04*	0.03	154
*East‐Qc*							
YA‐1,2	1*	—	—	—	—	—	4
RI‐3,4	1*	—	—	—	—	—	1
SF‐2	—	1*	—	—	—	—	4
SF‐4,5	1	—	—	—	—	—	4
SF‐10	1	—	—	—	—	—	2
SF‐12	1*	—	—	—	—	—	28
SF‐14	0.89*	—	—	0.11	—	—	5
Total	0.55*+0.29	0.14*	—	0.02	—	—	48

## 4 DISCUSSION

### 4.1 Diversity of cybrids

The diversity of *C. neogaeus* mitochondrial DNA detected in *C. eos* cybrids individuals revealed multiple mitochondrial introgression events. A comparison of these different haplotypes with sympatric hybrid lineages revealed two distinct histories of mitochondrial introgression.

We first confirmed that the cybrids had been independently produced at several sites during the Holocene. The correspondence of haplotypes between cybrids and sympatric hybrids suggests seven de novo transfers of *C. neogaeus* mtDNA to *C. eos*. In West‐Qc, cybrids carrying B III haplotype were detected in two geographically close sites. As we cannot rule out the migration of cybrids from one site to another, this result suggests at least one occurrence. In East‐Qc, *C. neogaeus* mtDNA transferred to *C. eos* differed among sites and originated from distinct hybrid lineages, supporting five distinct introgression events. While cybrids from sites YA‐1,2 and RI‐3,4 displayed the same sequence, the distinct composition of hybrids did not supported migration between those sites. As hybrids of this region occurred in situ (Vergilino et al., 2016), we can conclude these transfers to *C. eos* also occurred during the Holocene.

However, five haplotypes detected in cybrids had no counterpart in sympatric hybrids and four of them displayed correspondence with none of the hybrids analyzed in this study. This could be the result of two nonmutually exclusive events. Firstly, cybrids may have been produced before the extinction of the hybrid lineage that gave birth to them or the hybrid is rare and has not been previously sampled. These hypotheses are likely for four of five haplotypes localized to a single or a few numbers of sites. A nonmutually exclusive hypothesis is that introgression events predated the end of the Pleistocene and cybrids colonized these sites during the postglacial expansion. This appears the most parsimonious scenario for cybrids with haplotype A IV as they were widespread across West‐Qc and account for 93% of the cybrids from this region, even in the absence of hybrids harboring this haplotype.

A comparison of the distribution of cybrids from the two surveyed regions revealed two contrasting pictures that strikingly paralleled those observed in *C. eos‐neogaeus* hybrids. Most of the cybrids from West‐Qc are expected to have originated from postglacial expansion. Postglacial colonization also appeared as the main source of hybrids, with one lineage colonizing the entire region (Angers & Schlosser, 2007; Vergilino et al., 2016). Colonization by a unique founder group for either hybrids or cybrids resulted in the low haplotypic diversity observed throughout the West‐Qc region. However, 69.86% of the cybrids in East‐Qc were produced in situ, as confirmed by the presence of hybrids with an identical haplotype. Most of the 36 hybrid lineages detected in East‐Qc also occurred in situ during the Holocene and displayed restricted distribution areas (Vergilino et al., 2016). This high diversity of hybrids geographically organized is also reflected in the locally produced cybrids.

### 4.2 Fitness of cybrids in native environment

As introgression events are expected to continuously occur via hybrids, this additional input of individuals can demographically favor cybrids. They have then the potential to exclude wild types even if both display similar fitness (Barron et al., 2016). One prediction is that most of the sites only harbored cybrids and hybrids. However, a striking result is that hybrids and cybrids did not coexist together without wild types. This leads us to propose two nonmutually exclusive hypotheses about the demography of cybrids.

A first hypothesis is that de novo production of cybrids did not frequently occur and does not represent a substantial contribution to the population growth of cybrids. The low abundance of cybrids in their native environment suggests that effective production of cybrids from hybrids is likely limited and only occasional. These empirical results strongly contrast with the expectations of Barron et al.'s theoretical model (2016). Under their model, an abundance of hybrids allowing the formation of cybrids as low as 5% is expected to generate high amount of cybrids, leading the sympatric wild type to extinction (Barron et al., 2016). In the current study, wild‐type *C. eos* and hybrids are found together in 13 different sites but only seven of them harbored locally produced cybrids. Moreover, we failed to detect genetic signature of de novo production of cybrids for eight hybrid lineages (Figure 3). Therefore, de novo production of cybrids is not only dependent on the presence of hybrids, as proposed by Barron et al. (2016), but also other factors, such as genetic, ecological, and environmental conditions, may hinder the efficiency of cybrid production.

The second hypothesis is that cybrids did not have higher fitness than wild types in their native environment. A mtDNA haplotype under positive selection is expected to increase in abundance and reach fixation (Smith & Haigh, 1974). In sites where in situ production of cybrids has been inferred, *C. eos* wild types were always present, indicating that in their native environment, cybrids could not exclude wild types. Furthermore, wild types were more abundant in East‐QC, where most of the de novo introgression events were observed. We can therefore conclude that allospecific mtDNA do not provide an immediate fitness advantage to locally produced cybrid. This prevents the competitive exclusion of wild types well adapted to these local conditions, even if cybrids can be occasionally produced by hybrids.

### 4.3 Fitness of colonizing cybrids

The postglacial origin of most of the cybrids from the West‐Qc sites provides a valuable system to assess their fitness across different environmental conditions. Cybrids and wild types were usually detected in allopatry in West‐Qc, and their distribution could not be explained by either geographic distance or hydrologic network. Such a distribution could be the result of random processes associated with postglacial dispersal or the genetic drift of mtDNA. However, we can rule out these hypotheses when taking the presence of hybrids into account. Because hybrids require the sperm of one closely related sexual species to reproduce (Goddard et al., 1998), they are expected to co‐occur as frequently with wild types as do cybrids in a null model of distribution. However, when co‐occurrences were computed and tested by permutation following the method described in Borcard, Gillet, and Legendre (2011), the results revealed that hybrids co‐occurred more frequently with wild types (*p* = .003) than with cybrids (*p* = .340). For instance, considering sites where wild types and cybrids where allopatric, hybrids coexisted with wild types in three of the six sites but occurred in none of the seven sites where only the cybrids were found. The strong co‐occurrence of wild type and hybrids coupled with the allopatry of cybrids make random processes unlikely to explain their respective distribution.

An alternative hypothesis is that wild types/hybrids and cybrids have colonized (or survive in) sites with different ranges of environmental conditions. In the absence of hybrids with haplotype A IV, we can rule out contemporaneous production of that cybrid in West‐Qc region. This indicated that cybrids may have higher fitness than wild types in a given range of environmental conditions. This does not contradict the hypothesis that cybrids did not have higher fitness than wild types in their native environment. For instance, the higher abundance of cybrids reported in the northern part of *C. eos* distribution led Mee & Taylor (2012) to suggest that *C. neogaeus* mitochondria provide a fitness advantage over the *C. eos* mitochondria in colder habitats. *Chrosomus neogaeus* mtDNA has a significant influence on the phenotype of individuals as different epigenomes and proteomes were detected between the wild types and cybrids (Angers, Dallaire, Vervaet, Vallieres, & Angers, 2012). Cybrids also present a higher mitochondrial respiratory chain complex IV activity and higher swimming capacity than the wild‐type *C. eos* (Deremiens et al., 2015). The hypothesis that cybrids may have a better fitness in specific conditions is therefore consistent with the literature reporting that mitochondrial introgression may allow one species to increase its range of distribution or its tolerance to environmental changes (Blier, Breton, Desrosiers, & Lemieux, 2006; Boratyński et al., 2011; Toews et al., 2014).

In conclusion, this study revealed the importance of analyzing multiple instantaneous introgression events as the fitness advantages associated with allospecific mtDNA may strongly differ between native environments and those colonized during the postglacial expansion. The allospecific mtDNA does not provide better fitness in the native environment of cybrids when compared to that of wild types expected to be well adapted to local conditions, making the success of an introgressed lineage uncertain. However, cybrids may have higher fitness than wild types in specific conditions and can allow for the colonization of environments different from those of the wild type, thus expanding the range of a species.

## CONFLICT OF INTEREST

None declared.

## DATA ACCESSIBILITY

DNA sequences: GenBank accessions MG793359 to MG793378. All relevant data are within the article.

## AUTHOR CONTRIBUTIONS

BA, CL, and RV designed the study. CL, RV, LD, and RV carried the laboratory work. BA and CL performed data analyses. All authors contributed to the manuscript and approved the final manuscript.

## References

[ece33922-bib-0001] Allendorf, F. W. , Leary, R. F. , Spruell, P. , & Wenburg, J. K. (2001). The problems with hybrids: Setting conservation guidelines. Trends in Ecology & Evolution, 16, 613–622.

[ece33922-bib-0002] Alves, P. C. , Melo‐Ferreira, J. , Freitas, H. , & Boursot, P. (2008). The ubiquitous mountain hare mitochondria: Multiple introgressive hybridization in hares, genus *Lepus* . Philosophical Transactions of the Royal Society of London, Series B: Biological Sciences, 363, 2831–2839. 18508749 10.1098/rstb.2008.0053 PMC2606744

[ece33922-bib-0003] Angers, B. , Dallaire, A. , Vervaet, S. , Vallieres, F. , & Angers, A. (2012). The influence of mitochondria in epigenetics revealed through naturally occurring fish cybrids. Current Zoology, 58, 138–145.

[ece33922-bib-0004] Angers, B. , & Schlosser, I. J. (2007). The origin of *Phoxinus eos‐neogaeus* unisexual hybrids. Molecular Ecology, 16, 4562–4571. 17892466 10.1111/j.1365-294X.2007.03511.x

[ece33922-bib-0005] April, J. , Hanner, R. H. , Dion‐Côté, A.‐M. , & Bernatchez, L. (2013). Glacial cycles as an allopatric speciation pump in north‐eastern American freshwater fishes. Molecular Ecology, 22, 409–422. 23206322 10.1111/mec.12116

[ece33922-bib-0006] Barron, J. N. , Lawson, T. J. , & Jensen, P. A. (2016). Analysis of potential factors allowing coexistence in a sexual/asexual minnow complex. Oecologia, 180, 707–715. 26650583 10.1007/s00442-015-3522-0

[ece33922-bib-0007] Bernatchez, L. , & Wilson, C. C. (1998). Comparative phylogeography of Nearctic and Palearctic fishes. Molecular Ecology, 7, 431–452.

[ece33922-bib-0008] Binet, M. C. , & Angers, B. (2005). Genetic identification of members of the *Phoxinus eos‐neogaeus* hybrid complex. Journal of Fish Biology, 67, 1169–1177.

[ece33922-bib-0009] Blier, P. U. , Breton, S. , Desrosiers, V. , & Lemieux, H. (2006). Functional conservatism in mitochondrial evolution: Insight from hybridization of arctic and brook charrs. Journal of Experimental Zoology Part B: Molecular and Developmental Evolution, 306, 425–432. 10.1002/jez.b.21089 16404737

[ece33922-bib-0010] Boratyński, Z. , Alves, P. C. , Berto, S. , Koskela, E. , Mappes, T. , & Melo‐Ferreira, J. (2011). Introgression of mitochondrial DNA among *Myodes* voles: Consequences for energetics? BMC Evolutionary Biology, 11, 355. 22151479 10.1186/1471-2148-11-355 PMC3260118

[ece33922-bib-0011] Borcard, D. , Gillet, F. , & Legendre, P. (2011). Numerical ecology with R. New York, NY: Springer Science Business & Media.

[ece33922-bib-0012] Burton, R. S. , Pereira, R. J. , & Barreto, F. S. (2013). Cytonuclear genomic interactions and hybrid breakdown. Annual Review of Ecology, Evolution, and Systematics, 44, 281–302.

[ece33922-bib-0013] Deremiens, L. , Schwartz, L. , Angers, A. , Glémet, H. , & Angers, B. (2015). Interactions between nuclear genes and a foreign mitochondrial genome in the redbelly dace *Chrosomus eos* . Comparative Biochemistry and Physiology. Part B, Biochemistry & Molecular Biology, 189, 80–86. 10.1016/j.cbpb.2015.08.002 26277640

[ece33922-bib-0014] Dowling, T. E. , & Secor, C. L. (1997). The role of hybridization in diversification of animals. Annual Review of Ecology & Systematics, 28, 593.

[ece33922-bib-0015] Edmands, S. , & Burton, R. S. (1999). Cytochrome c oxidase activity in interpopulation hybrids of a marine copepod: A test for nuclear‐nuclear or nuclear‐cytoplasmic coadaptation. Evolution, 53, 1972–1978. 28565439 10.1111/j.1558-5646.1999.tb04578.x

[ece33922-bib-0016] Ellison, C. K. , & Burton, R. S. (2008). Genotype‐dependent variation of mitochondrial transcriptional profiles in interpopulation hybrids. Proceedings of the National Academy of Sciences of the United States of America, 105, 15831–15836. 18843106 10.1073/pnas.0804253105 PMC2572918

[ece33922-bib-0017] Gagnon, M. C. , & Angers, B. (2006). The determinant role of temporary proglacial drainages on the genetic structure of fishes. Molecular Ecology, 15, 1051–1065. 16599966 10.1111/j.1365-294X.2005.02828.x

[ece33922-bib-0018] Goddard, K. A. , Dawley, R. M. , & Dowling, T. E. (1989). Origin and genetic relationships of diploid, triploid, and diploid‐triploid mosaic biotypes in the *Phoxinus eos‐neogaeus* unisexual complex In Dawley R., & Bogart J. (Eds.), Evolution and ecology of unisexual vertebrates (pp. 268–280). New York, NY: New York State Museum.

[ece33922-bib-0019] Goddard, K. , Megwinoff, O. , Wessner, L. , & Giaimo, F. (1998). Confirmation of gynogenesis in *Phoxinus eos‐neogaeus* (Pisces: Cyprinidae). Journal of Heredity, 89, 151–157.

[ece33922-bib-0020] Goddard, K. , & Schultz, R. (1993). Aclonal reproduction by polyploid members of the clonal hybrid species *Phoxinus eos‐neogaeus* (Cyprinidae). Copeia, 3, 650–660.

[ece33922-bib-0021] Goudet, J. (2001). FSTAT, a program to estimate and test gene diversities and fixation indices (version 2.9.3). Department of Ecology and Evolution, University of Lausanne, Switzerland. Retrieved from http://www2.unil.ch/popgen/softwares

[ece33922-bib-0022] Holm, S. (1979). A simple sequentially rejective multiple test procedure. Scandinavian Journal of Statistics, 6, 65–70.

[ece33922-bib-0023] Irwin, D. E. , Rubtsov, A. S. , & Panov, E. N. (2009). Mitochondrial introgression and replacement between yellowhammers (*Emberiza citrinella*) and pine buntings (*Emberiza leucocephalos*)(Aves: Passeriformes). Biological Journal of the Linnean Society, 98, 422–438.

[ece33922-bib-0024] Jonsson, B. G. (2001). A null model for randomization tests of nestedness in species assemblages. Oecologia, 127, 309–313. 28547100 10.1007/s004420000601

[ece33922-bib-0025] Kumar, S. , Stecher, G. , & Tamura, K. (2016). MEGA7: Molecular Evolutionary Genetics Analysis version 7.0 for bigger datasets. Molecular Biology and Evolution, 33, 1870–1874. 27004904 10.1093/molbev/msw054 PMC8210823

[ece33922-bib-0026] Le Bras, M. , Clément, M. , Pervaiz, S. , & Brenner, C. (2005). Reactive oxygen species and the mitochondrial signaling pathway of cell death. Histology and Histopathology, 20, 205. 15578439 10.14670/HH-20.205

[ece33922-bib-0027] Leung, C. , & Angers, B. (2018). Imitating the cost of males: A hypothesis for coexistence of all‐female sperm‐dependent species and their sexual host. Ecology and Evolution, 8, 266–272. 29321869 10.1002/ece3.3681 PMC5756870

[ece33922-bib-0028] Leung, C. , Breton, S. , & Angers, B. (2016). Facing environmental predictability with different sources of epigenetic variation. Ecology and Evolution, 6, 5234–5245. 27551379 10.1002/ece3.2283 PMC4984500

[ece33922-bib-0029] Lewontin, R. C. , & Birch, L. C. (1966). Hybridization as a source of variation for adaptation to new environments. Evolution, 20, 315–336. 28562982 10.1111/j.1558-5646.1966.tb03369.x

[ece33922-bib-0030] Liu, K. , Wang, F. , Chen, W. , Tu, L. , Min, M. S. , Bi, K. , & Fu, J. (2010). Rampant historical mitochondrial genome introgression between two species of green pond frogs, *Pelophylax nigromaculatus* and *P. plancyi* . BMC Evolutionary Biology, 10, 201. 20587049 10.1186/1471-2148-10-201 PMC2909235

[ece33922-bib-0031] Llopart, A. , Herrig, D. , Brud, E. , & Stecklein, Z. (2014). Sequential adaptive introgression of the mitochondrial genome in *Drosophila yakuba* and *Drosophila santomea* . Molecular Ecology, 23, 1124–1136. 24460929 10.1111/mec.12678 PMC4260671

[ece33922-bib-0032] Marková, S. , Dufresne, F. , Manca, M. , & Kotlík, P. (2013). Mitochondrial capture Misleads about ecological speciation in the *Daphnia pulex* complex. PLoS ONE, 8, e69497. 23869244 10.1371/journal.pone.0069497 PMC3711805

[ece33922-bib-0033] Mavárez, J. , & Linares, M. (2008). Homoploid hybrid speciation in animals. Molecular Ecology, 17, 4181–4185. 19378399 10.1111/j.1365-294x.2008.03898.x

[ece33922-bib-0034] McGuire, J. A. , Linkem, C. W. , Koo, M. S. , Hutchison, D. W. , Lappin, A. K. , Orange, D. I. , … Jaeger, J. R. (2007). Mitochondrial introgression and incomplete lineage sorting through space and time: Phylogenetics of crotaphytid lizards. Evolution, 61, 2879–2897. 17941840 10.1111/j.1558-5646.2007.00239.x

[ece33922-bib-0035] McKenzie, M. , Chiotis, M. , Pinkert, C. A. , & Trounce, I. A. (2003). Functional respiratory chain analyses in murid xenomitochondrial cybrids expose coevolutionary constraints of cytochrome b and nuclear subunits of complex III. Molecular Biology and Evolution, 20, 1117–1124. 12777531 10.1093/molbev/msg132

[ece33922-bib-0036] Mee, J. , & Taylor, E. (2012). The cybrid invasion: Widespread postglacial dispersal by *Phoxinus* (Pisces: Cyprinidae) cytoplasmic hybrids. Canadian Journal of Zoology, 90, 577–584.

[ece33922-bib-0037] Montooth, K. L. , Meiklejohn, C. D. , Abt, D. N. , & Rand, D. M. (2010). Mitochondrial–nuclear epistasis affects fitness within species but does not contribute to fixed incompatibilities between species of *Drosophila* . Evolution, 64, 3364–3379. 20624176 10.1111/j.1558-5646.2010.01077.x PMC2997886

[ece33922-bib-0038] Muhlfeld, C. C. , Kalinowski, S. T. , McMahon, T. E. , Taper, M. L. , Painter, S. , Leary, R. F. , & Allendorf, F. W. (2009). Hybridization rapidly reduces fitness of a native trout in the wild. Biology Letters, 5, 328–331. 19324629 10.1098/rsbl.2009.0033 PMC2679930

[ece33922-bib-0039] New, J. G. (1962). Hybridization between two cyprinids, *Chrosomus eos* and *Chrosomus neogaeus* . Copeia, 1962, 147–152.

[ece33922-bib-0040] Oksanen, J. , Blanchet, F. G. , Kindt, R. , Legendre, P. , O'Hara, R. B. , Simpson, G. L. , … Wagner, H. (2015). vegan: Community ecology package. Retrieved from http://CRAN.R-project.org/package=vegan

[ece33922-bib-0041] Orita, M. , Suzuki, Y. , Sekiya, T. , & Hayashi, K. (1989). Rapid and sensitive detection of point mutations and DNA polymorphisms using the polymerase chain reaction. Genomics, 5, 874–879. 2687159 10.1016/0888-7543(89)90129-8

[ece33922-bib-0042] Osada, N. , & Akashi, H. (2012). Mitochondrial‐nuclear interactions and accelerated compensatory evolution: Evidence from the primate cytochrome c oxidase complex. Molecular Biology and Evolution, 29, 337–346. 21890478 10.1093/molbev/msr211

[ece33922-bib-0043] R Development Core Team (2004). R: A language and environment for statistical computing. Vienna, Austria: R Foundation for Statistical Computing.

[ece33922-bib-0044] Rand, D. M. , Fry, A. , & Sheldahl, L. (2006). Nuclear–mitochondrial epistasis and Drosophila aging: Introgression of *Drosophila simulans* mtDNA modifies longevity in *D. melanogaster* nuclear backgrounds. Genetics, 172, 329–341. 16219776 10.1534/genetics.105.046698 PMC1456161

[ece33922-bib-0045] Rheindt, F. E. , & Edwards, S. V. (2011). Genetic introgression: An integral but neglected component of speciation in birds. The Auk, 128, 620–632.

[ece33922-bib-0046] Rhymer, J. M. , & Simberloff, D. (1996). Extinction by hybridization and introgression. Annual Review of Ecology and Systematics, 27, 83–109.

[ece33922-bib-0147] Rieseberg, LH. , Wendel, JF. (1993). Introgression and its consequences in plants. In: Hybrid zones and the evolutionary process.(ed. Harrison RG), pp. 70‐109. Oxford University Press, Oxford, UK.

[ece33922-bib-0047] Ruedi, M. , Smith, M. , & Patton, J. (1997). Phylogenetic evidence of mitochondrial DNA introgression among pocket gophers in New Mexico (family Geomyidae). Molecular Ecology, 6, 453–462. 9161013 10.1046/j.1365-294x.1997.00210.x

[ece33922-bib-0048] Seehausen, O. (2006). Conservation: Losing biodiversity by reverse speciation. Current Biology, 16, R334–R337. 16682344 10.1016/j.cub.2006.03.080

[ece33922-bib-0049] Smith, J. M. , & Haigh, J. (1974). The hitch‐hiking effect of a favourable gene. Genetics Research, 23, 23–35. 4407212

[ece33922-bib-0050] Stebbins, G. L. (1959). The role of hybridization in evolution. Proceedings of the American Philosophical Society, 103, 231–251.

[ece33922-bib-0051] Stebbins, G. L. (1985). Polyploidy, hybridization, and the invasion of new habitats. Annals of the Missouri Botanical Garden, 72, 824–832.

[ece33922-bib-0052] Stone, L. , & Roberts, A. (1990). The checkerboard score and species distributions. Oecologia, 85, 74–79. 28310957 10.1007/BF00317345

[ece33922-bib-0053] Toews, D. P. , & Brelsford, A. (2012). The biogeography of mitochondrial and nuclear discordance in animals. Molecular Ecology, 21, 3907–3930. 22738314 10.1111/j.1365-294X.2012.05664.x

[ece33922-bib-0054] Toews, D. P. , Mandic, M. , Richards, J. G. , & Irwin, D. E. (2014). Migration, mitochondria, and the yellow‐rumped warbler. Evolution, 68, 241–255. 24102562 10.1111/evo.12260

[ece33922-bib-0055] Vergilino, R. , Leung, C. , & Angers, B. (2016). Inconsistent phylogeographic pattern between a sperm dependent fish and its host: In situ hybridization vs dispersal. BMC Evolutionary Biology, 16, 1–12. 27600616 10.1186/s12862-016-0754-5 PMC5012089

[ece33922-bib-0056] Wang, B. , & Wang, X.‐R. (2014). Mitochondrial DNA capture and divergence in Pinus provide new insights into the evolution of the genus. Molecular Phylogenetics and Evolution, 80, 20–30. 25106134 10.1016/j.ympev.2014.07.014

[ece33922-bib-0057] Wilson, C. C. , & Bernatchez, L. (1998). The ghost of hybrids past: Fixation of arctic charr (*Salvelinus alpinus*) mitochondrial DNA in an introgressed population of lake trout (*S. namaycush*). Molecular Ecology, 7, 127–132.

[ece33922-bib-0058] Yamada, A. , Kodo, Y. , Murakami, M. , Kuroda, M. , Aoki, T. , Fujimoto, T. , & Arai, K. (2015). Hybrid origin of gynogenetic clones and the introgression of their mitochondrial genome into sexual diploids through meiotic hybridogenesis in the loach, *Misgurnus anguillicuadatus* . Journal of Experimental Zoology Part A: Ecological Genetics and Physiology, 323, 593–606. 10.1002/jez.1950 26173834

[ece33922-bib-0059] Zieliński, P. , Nadachowska‐Brzyska, K. , Wielstra, B. , Szkotak, R. , Covaciu‐Marcov, S. D. , Cogălniceanu, D. , & Babik, W. (2013). No evidence for nuclear introgression despite complete mtDNA replacement in the Carpathian newt (*Lissotriton montandoni*). Molecular Ecology, 22, 1884–1903. 23379646 10.1111/mec.12225

